# Decrease of Medical Students

**Published:** 1855-04

**Authors:** 


*Decrease of Medical Students.*

WE cannot yet gather from our exchanges sufficient returns to enable us to present a table, showing either the whole number of Students or Graduates, who have been in attendance at the various Medical Colleges of our country during the past winter. Enough, however, has been published to show that the gradual decrease of both which has been taking place during the last three or four years, has been exhibited in a much more marked degree during the past winter than at any time before. But it is easy to perceive a marked difference in the ratio of diminution in the different colleges. Those located in our large commercial cities, with good advantages for clinical or hospital instruction, have, as a general rule, retained nearly their usual number, and in the Rush Medical College, of our own city, the number of graduates has actually increased. But in most of the colleges located in villages and rural districts, the diminution of numbers in attendance has been very great. This is as it should be, and indicates the operation of an influence which is destined to induce ultimately a most salutary change in our system of medical education. Not a few have hastily proclaimed the American Medical Association a failure, and its influence over the Medical Colleges a nullity. Such have looked only at the surface of things. While it is true that neither the profession generally, nor the colleges have formally adopted and carried out the recommendations of the Association in regard to preliminary, college, and clinical instruction, a little examination will show that the influence actually exerted on all these topics is decided and salutary. We are certain, from our intercourse with both practitioners and students, that the former are much more cautious than formerly about receiving into their offices young men who have no preliminary education whatever; and the latter are much more generally aware of the necessity of a higher grade of intellectual cultivation to ensure success in the profession. Other circumstances have, doubtless, exerted more or less influence on the number engaging in the study of medicine; but the increased attention excited throughout the whole profession by the annual discussions in the meetings of the Association, has accomplished far more than any other one cause. So, too, the position taken by the Association in regard to the great importance of true hospital clinical instruction, has been the chief cause of the greater relative concentration of students in those schools where clinical instruction in hospitals can be enjoyed.

So true is this, that should the same ratio of decrease in the number of students attending many of the *country* schools, continue one or two years longer they would be obliged to suspend altogether. The influence of the Association over the Medical colleges in another important particular, is already visible. Previous to the existence of the National Organization, there was no mode by which the most active, working members of the profession could be brought in contact with, and made to know each other. Hence there was a constant tendency on the part of the colleges, to choose men to fill whatever vacancies might occur in their respective faculties from a narrow circles of friends and alumni. The annually recurring meetings of the National Association are rapidly correcting this evil, by bringing the most active, industrious, and talented members of the profession in all sections of our widely extended country, not only in contact with each other but also prominently before the whole profession. It is thus that the profession becomes nationalized, and the influence of mere sectional cliques destroyed. Let the same tendency be kept up by sustaining the National Association, and all the colleges will soon be compelled by a just professional sentiment, to choose men to fill the vacancies which will occur from time to time in their faculties, from among the most talented and best qualified, without reference to the section or locality from which they may come.

